# *C9ORF72* hexanucleotide repeat exerts toxicity in a stable, inducible motor neuronal cell model, which is rescued by partial depletion of Pten

**DOI:** 10.1093/hmg/ddx022

**Published:** 2017-02-01

**Authors:** Matthew J. Stopford, Adrian Higginbottom, Guillaume M. Hautbergue, Johnathan Cooper-Knock, Padraig J. Mulcahy, Kurt J. De Vos, Alan E. Renton, Hannah Pliner, Andrea Calvo, Adriano Chio, Bryan J. Traynor, Mimoun Azzouz, Paul R. Heath, ITALSGEN Consortium, NeuroX Consortium, Janine Kirby, Pamela J. Shaw

**Affiliations:** 1 Department of Neuroscience, Sheffield Institute for Translational Neuroscience, University of Sheffield, Sheffield S10 2HQ, UK; 2 Neuromuscular Diseases Research Section, National Institute on Aging, National Institutes of Health, Bethesda, MD 20892, USA; 3 Department of Neuroscience, University of Turin, Turin, Italy

## Abstract

Amyotrophic lateral sclerosis (ALS) is a devastating and incurable neurodegenerative disease, characterised by progressive failure of the neuromuscular system. A (G4C2)n repeat expansion in *C9ORF72* is the most common genetic cause of ALS and frontotemporal dementia (FTD). To date, the balance of evidence indicates that the (G4C2)n repeat causes toxicity and neurodegeneration via a gain-of-toxic function mechanism; either through direct RNA toxicity or through the production of toxic aggregating dipeptide repeat proteins. Here, we have generated a stable and isogenic motor neuronal NSC34 cell model with inducible expression of a (G4C2)102 repeat, to investigate the gain-of-toxic function mechanisms. The expression of the (G4C2)102 repeat produces RNA foci and also undergoes RAN translation. In addition, the expression of the (G4C2)102 repeat shows cellular toxicity. Through comparison of transcriptomic data from the cellular model with laser-captured spinal motor neurons from C9ORF72-ALS cases, we also demonstrate that the PI3K/Akt cell survival signalling pathway is dysregulated in both systems. Furthermore, partial knockdown of Pten rescues the toxicity observed in the NSC34 (G4C2)102 cellular gain-of-toxic function model of C9ORF72-ALS. Our data indicate that PTEN may provide a potential therapeutic target to ameliorate toxic effects of the (G4C2)n repeat.

## Introduction

A (G4C2)n repeat expansion in a non-coding region of the C9ORF72 gene has been established as the most common identified genetic cause of amyotrophic lateral sclerosis (ALS) as well as frontotemporal dementia (FTD) (1,2). Expansions of  >30 repeats are considered pathogenic (3,4), but expansions of 200–5000 repeats are more commonly detected in ALS patients (5). The mechanism(s) by which the repeat causes neuronal death in the motor cortex, brainstem and spinal cord in ALS and/or neuronal death in the frontal and temporal lobes of the brain in FTD are currently being elucidated, with three hypotheses proposed which are not mutually exclusive: (1) Haploinsufficiency of C9ORF72; (2) RNA toxicity; and (3) Dipeptide repeat protein (DPR) toxicity.

Various reports demonstrate *C9ORF72* mRNA is reduced in post-mortem CNS tissue, lymphoblast cells and iPSC-derived neurons of patients containing the (G4C2)n repeat expansion (1,6–10) and that C9ORF72 protein is also reduced in the frontal cortex of patients with the repeat expansion (10,11), suggesting C9ORF72 haploinsufficiency as a potential pathogenic mechanism. In addition, knockdown or deletion of C9ORF72 orthologues in zebrafish and *CaenorhabditisElegans*, respectively, caused motor dysfunction (7,12). C9ORF72 protein regulates autophagy induction and autophagic flux, and both C9ORF72-ALS/FTD derived neurons and mice lacking functional C9ORF72 protein have impaired autophagy (13–15). However, mice lacking functional C9ORF72 do not show signs of neurodegeneration or reduced motor function, although they do display autoimmunity and other immune system dysregulation (13,16–19). Equally, when C9ORF72 knockdown (either partial or complete) was confined to the CNS in adult mice, there were no behavioural or motor defects, nor any signs of neuropathology associated with ALS and FTD (20,21).

The (G4C2)n repeat expansion is also suggested to exert a toxic gain-of-function via either direct RNA toxicity and/or DPR proteins. The (G4C2)n repeat is transcribed in both sense and antisense directions, and forms sense (G4C2)n and antisense (C4G2)n RNA foci, respectively. These RNA foci are present in C9ORF72-ALS/FTD CNS tissue and neuronal cells (1,22), and may potentially sequester essential RNA binding proteins leading to dysregulated RNA processing, as seen in other repeat expansion disorders (23). Several RNA binding proteins bind (G4C2)n and/or (C4G2)n RNA *in vitro*, and also colocalise with RNA foci in patient CNS tissue (9,24–29). In addition, transcriptomic analysis of C9ORF72-ALS/FTD patient CNS tissue showed dysregulation in RNA splicing and processing (30,31).

The (G4C2)n repeat also undergoes unconventional repeat associated non-ATG (RAN) translation in all reading frames in both the sense and antisense directions, forming aggregation-prone DPR proteins (32,33). The five species of DPR proteins (poly-GA, poly-GR, poly-GP, poly-AP and poly-PR) produced form insoluble inclusions in C9ORF72-ALS/FTD patient CNS tissue (32–35). DPR proteins are toxic in cultured cells and cause neurodegeneration in *Drosophila* models (35–42). The arginine-rich DPR proteins (poly-GR and poly-PR) appear particularly toxic, localise to the nucleolus, disrupt ribosomal RNA biogenesis and cause cell death (36–39,41). Also, in two elegant studies using *Drosophila* models, the toxicity of (G4C2)n repeats was dependent on the production of DPR, and not (G4C2)n RNA foci (41,43). However, while these studies suggest that the DPR are the likely major toxic insult derived from the sense (G4C2)n RNA, antisense (C4G2)n RNA foci, but not sense (G4C2)n RNA foci, correlate with TDP-43 proteinopathy in motor neurons from C9ORF72-ALS patients (44). Furthermore in C9ORF72-ALS patients, DPR load is much lower in spinal motor neurons compared with other unaffected regions of CNS, and TDP-43 inclusions rarely co-localise with DPR suggesting that they may not be the primary toxic insult in motor neuron degeneration (45–48).

Several recently generated mouse models of C9ORF72-ALS have produced fairly variable results. In one study, a (G4C2)66 construct was delivered to the CNS of the mice and resulted in RNA foci, DPR, and TDP-43 pathology, as well as behavioural and motor defects (49). In two other studies, C9ORF72 BAC transgenic mouse models were generated that contain the (G4C2)n repeat expansion within either part of or all of the C9ORF72 gene and display both the RNA foci and DPRs, yet surprisingly did not develop signs of neurodegeneration or ALS/FTD phenotypes (50,51). Another group described similar findings in different C9ORF72 BAC lines, with the addition of a cognitive phenotype (52). However, in a fourth C9ORF72 BAC mouse model, there was TDP-43 pathology, motor neuron degeneration and a neurodegenerative phenotype, including weakness, weight loss, breathing problems and decreased survival, as well as anxiety-like behaviour (53). While these studies are inconsistent in their findings, they demonstrate that (G4C2)n repeat length and expression level, as well as other contributing genetic factors may contribute to ALS pathogenesis.

In further support of a gain-of-toxic function, ASOs that target sense *C9ORF72* transcripts not only reduce (G4C2)n RNA foci number, they also ameliorate transcriptomic changes and reduce toxicity in iPSC-derived neuronal cells from C9ORF72-ALS/FTD patients (9,20,27). The balance of evidence is emerging that a gain-of-toxic function is more likely than C9ORF72 haploinsufficiency to provide the major toxic insult that drives C9ORF72-ALS and/or FTD. However, the relative contributions of the sense and antisense RNA, and each of the DPR species in the neuronal injury of C9ORF72-ALS and FTD have not been fully established. In addition, a loss of C9ORF72 function may still exacerbate the primary toxic insult, contributing to ALS/FTD pathogenesis. Therefore, we aimed to generate a gain-of-toxic function model to identify potential therapeutic targets for C9ORF72-ALS. We generated a stable motor neuron-like cell model with inducible (G4C2)n expression, which allowed identification of the early biochemical changes associated with the expression of the (G4C2)n repeat expansion. Our results showed that (G4C2)102 constructs produced RNA foci, underwent RAN translation and caused toxicity in NSC34 cells. Transcriptomic analysis of the NSC34 cell model identified dysregulation in the PI3K/Akt signalling pathway, which was validated in motor neurons from C9ORF72-ALS patients. Further, we showed that partial knockdown of *Pten*, the normal function of which is to negatively regulate the PI3kinase/Akt cell survival pathway, provided a rescue effect in the NSC34 cell model, suggesting that PTEN could represent a potential therapeutic target for C9ORF72-ALS.

## Results

### Generation of stable NSC34 cell models with tetracycline-inducible (G4C2)n expression

**Figure 1:**
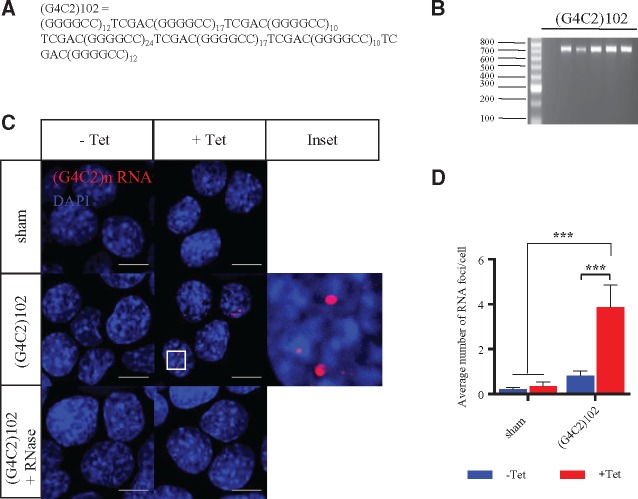
NSC34 (G4C2)102 cells have tetracycline inducible (G4C2)102 RNA expression, which forms RNA foci. **(A)** Sequence of the (G4C2)102 construct. **(B)** Gel electrophoresis sizing the (G4C2)102 repeat constructs in the pcDNA5/FRT/TO-(G4C2)102 plasmids. **(C)** NSC34 sham and NSC34 (G4C2)102 cells were cultured for 3 days with (or without) 0.5 µg/ml tetracycline. NSC34 (G4C2)102 cells were additionally treated with RNAse A as a control. Cells were stained with a locked nucleic acid (C4G2)3 sense probe (Red) and Dapi (Blue). Foci magnified 5× inset. Scale bar = 10 µm. **(D)** Average number of RNA foci per cell (***p < 0.001; two-way ANOVA with Tukey’s multiple comparisons post hoc test; data shown are mean and SD; *n* = 3).

The primary aim of this work was to establish the gain-of-function effects from the (G4C2)n repeat expansion independent of the C9ORF72 gene context, and thereby identify potential therapeutic targets for C9ORF72-ALS. To address this, we generated stable, isogenic motor neuronal NSC34 cell models with tetracycline-inducible expression of 0 (sham) or 102 (G4C2)n repeats. First, (G4C2)10 repeat constructs were synthesised, and subsequently used to generate interrupted (G4C2)n repeat constructs containing 102 repeats (Fig. 1A). These (G4C2)102 constructs were then sub-cloned into pcDNA5/FRT/TO plasmids (Invitrogen) that contain an FRT site and a CMV/tetracycline operator hybrid promoter (Fig. 1B). The pcDNA5/FRT/TO-(G4C2)102 vectors were then stably integrated into Flp-In™ T-REx™ NSC34 cells (that were generated in house). In addition, empty pcDNA5/FRT/TO vectors were integrated into Flp-In™ T-REx™ NSC34 cells, generating NSC34 sham as a control *in vitro* model.

The interrupted (G4C2)n repeat RNA forms characteristic RNA foci comparable to those detectable in the CNS of C9ORF72-ALS cases. Therefore, RNA fluorescence *in situ* hybridisation (FISH) was used to detect expression of the (G4C2)n RNA in the NSC34 (G4C2)102 cells. The number of sense (G4C2)n RNA foci increased when the cells were induced with tetracycline (Fig. 1C and D). In addition, most RNA foci were nuclear, but occasional cytoplasmic (G4C2)n RNA foci were also detected (Fig. 1C). RNA foci were not detected in NSC34 sham cells (Fig. 1C). In addition, RNAse A treatment ablated foci in the NSC34 (G4C2)102 cells (Fig. 1C).

**Figure 2:**
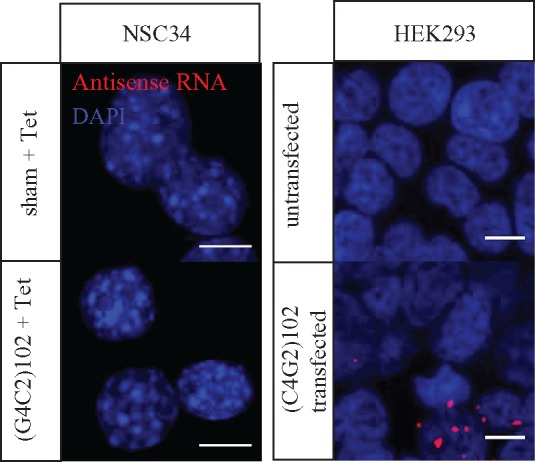
NSC34 (G4C2)n cells do not transcribe (G4C2)n in the antisense direction. NSC34 sham and NSC34 (G4C2)102 cells were cultured for 3 days with 0.5 µg/ml tetracycline. Cells were stained with a locked nucleic acid (G4C2)3 antisense probe (Red) and Dapi (Blue). HEK293 cells transfected with an antisense (C4G2)102 plasmid contain (C4G2)102 foci and serve as a positive control. Scale bar = 10 µm.

The NSC34 (G4C2)102 cells were also examined for antisense (C4G2)n RNA transcripts, using an antisense RNA FISH probe. As expected, no antisense (C4G2)n RNA foci were detected in the NSC34 (G4C2)102 cells (Fig. 2), because the (G4C2)n repeat is independent of the genomic C9ORF72 gene and any associated promoter(s) that drive transcription in the antisense direction. To demonstrate that we can detect antisense (C4G2)n RNA foci, we transfected antisense (C4G2)102 constructs into HEK293 cells, as a positive control (Fig. 2).

**Figure 3:**
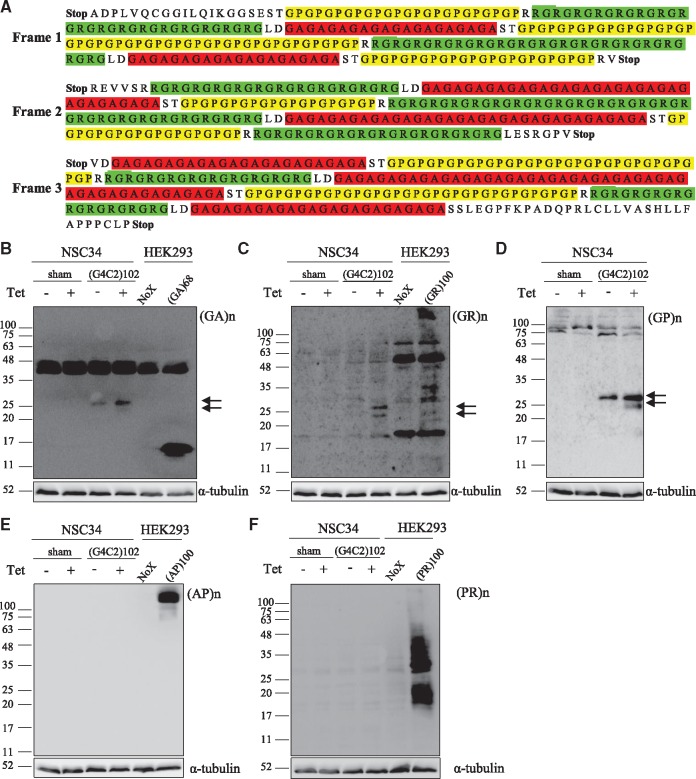
The (G4C2)102 construct undergoes RAN translation in the NSC34 (G4C2)102 (G4C2)102 cells. (**A**) Schematic of translation products (including Stop codons) from interrupted (G4C2)102 RNA construct in all three reading frames. GA, GP and GR repeats are highlighted in red, yellow and green, respectively. (**B–F**) NSC34 sham and NSC34 (G4C2)102 cells were cultured for 7 days with (or without) 0.5 µg/ml tetracycline. HEK293 cells were transfected with (GA)68, (GR)100, (AP)100 and (PR)100 constructs to serve as a positive control for the respective DPR antibody. Cells were lysed and immunoblotted with anti-poly-GA (B), anti-poly-GR (C), anti-poly-GP (D), anti-poly-AP (E) and anti-poly-PR (F). Arrows indicate the RAN translation products in the NSC34 (G4C2)102 cells. α-Tubulin was used as a loading control for blots. Molecular weight markers are indicated (kDa).

In addition, immunoblotting was performed to detect translation products of the (G4C2)102 RNA. Translation of the (G4C2)102 was predicted to change frame between DPR motifs at each TCGAC interruption, and, therefore, translation products would contain a mixture of poly-GA, poly-GR, and poly-GP stretches (Fig. 3A). NSC34 (G4C2)102 cells were immunoblotted using anti-poly-GA, anti-poly-GR, anti-poly-GP, anti-poly-AP and anti-poly-PR (Fig. 3B–F). HEK293 cells were transfected with (GA)68, (GR)100, (AP)100 and (PR)100 constructs, and used as positive controls for the respective antibodies (Fig. 3). There was no available positive control for the anti-poly-GP antibody. The anti-poly-GA, anti-poly-GR and anti-poly-GP antibodies all detected proteins at 24 and 27 kDa in the NSC34 (G4C2)102 cells treated with tetracycline (Fig. 3B–D), and these proteins were more abundant in the tetracycline-treated NSC34 (G4C2)102 compared with untreated NSC34 (G4C2)102 cells (Fig. 3B–D). In addition, these proteins were not detected in NSC34 sham, indicating the (G4C2)102 RNA was translated to produce proteins containing all three sense DPR motifs. Also, we concluded the (G4C2)102 RNA was translated via RAN translation as there are no ATG start sites preceding the (G4C2)102 construct in any frame (Fig. 3A). Further, poly-AP and poly-PR are specifically translated from the antisense (C4G2)n RNA, and neither anti-poly-AP nor anti-poly-PR detected any proteins specific to the NSC34 (G4C2)102 cells compared with NSC34 sham cells (Fig. 3E and F), supporting an absence of anti-sense (C4G2)n RNA transcription and subsequent translation. Finally, translation products were also detected using the anti-poly-GA, anti-poly-GR and anti-poly-GP antibodies in HEK293 cells containing the same interrupted (G4C2)102 repeat construct (data not shown).

### NSC34 (G4C2)102 cells recapitulate certain aspects of ALS pathology

**Figure 4:**
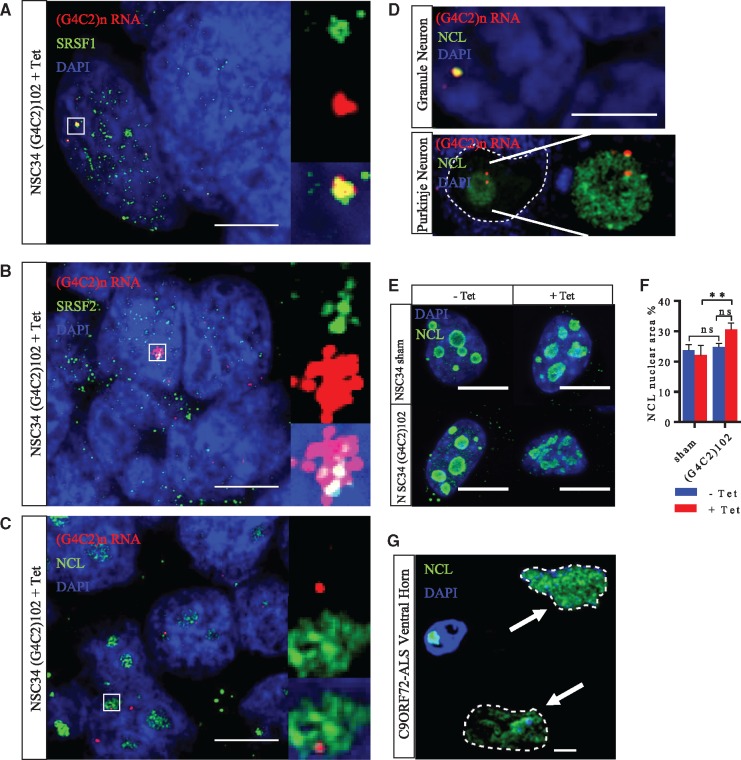
NSC34 (G4C2)102 cells recapitulate pathological features of C9ORF72-ALS. **(A–C, E)** NSC34 (G4C2)102 cells were induced with 0.5 µg/ml tetracycline for 5 days. Cells were then stained with a locked nucleic acid (C4G2)3 sense probe (Red), anit-SRSF1 (A), anti-SRSF2 (B) or anti-NCL (C) (all green), and Dapi (Blue). Scale bar = 10 µm. **(D)** Cerebellar slices from C9ORF72-ALS cases were stained with a locked nucleic acid (C4G2)3 sense probe (Red), anti-NCL (green) and Dapi (Blue). Scale bar = 3 µm. (E) NSC34 sham and NSC34 (G4C2)102 cells were stained for NCL (green) and DAPI (blue). Scale bar = 10 µm. **(F)** Quantification of NCL area as a percentage of nuclear area in NSC34 sham and (G4C2)102 cells (*p < 0.05; **p < 0.01; two-way ANOVA with Tukey’s multiple comparisons post hoc test; *n* = 3). **(G)** Ventral horn from C9ORF72-ALS was stained for NCL and DAPI. Motor neurons (indicated with arrows and nuclei outlined) show disrupted NCL staining, while glial cells do not show disrupted NCL staining. Scale bar = 3 µm.

To validate the NSC34 (G4C2)102 cells as a model of C9ORF72-ALS, they were assessed for biochemical changes that are relevant to the pathophysiology of C9ORF72-ALS. First, we and others have previously shown that several RNA binding proteins (RBPs) such as Serine/Arginine-Rich Splicing Factor 1 (SRSF1), Serine/Arginine-Rich Splicing Factor 2 (SRSF2) and Nucleolin (NCL) bind (G4C2)n RNA, and co-localise with (G4C2)n RNA foci in C9ORF72-ALS cells (24,28). Tetracycline-induced NSC34 (G4C2)102 cells were co-stained for sense (G4C2)n RNA foci as well as either SRSF1, SRSF2 or NCL (Fig. 4A–C). We analysed 50 cells for each RBP co-stain. Of the (G4C2)n RNA foci counted, 19.5% co-localised with SRSF1 puncta; 11.9% co-localised with SRSF2 puncta; and 22.6% co-localized with nucleolar NCL (Fig. 4A–C). We have previously shown (G4C2)n RNA foci co-localisation with SRSF2 in cerebellar granule cells and motor neurons in C9ORF72-ALS patients (24). NCL was also observed to co-localise with foci in cerebellar granule and Purkinje neurons from C9ORF72-ALS patients (Fig. 4D).

Second, nucleolar stress is implicated in C9ORF72-ALS pathology (28). Here we detected a degree of disruption to the nucleolar morphology, in the NSC34 (G4C2)102 cells (Fig. 4E). The nucleolar area as a percentage of the nucleus was increased by 38.6 ± 9.8% (*p* < 0.01) in the NSC34 (G4C2)102 + tet vs. NSC34 sham cells + tet (Fig. 4E and F). There was no difference in the overall nuclear area between the cell groups (data not shown). This effect was much more apparent in motor neurons from the ventral horn, but not glial cells, from C9ORF72-ALS patients (Fig. 4G). To establish if the wider ALS pathological features of TDP-43 mislocalisation or oxidative stress were also present in this cellular model, immunofluorescence microscopy and dichlorofluorescein (DCF) assays were performed on the NSC34 (G4C2)n cells. However, there was no TDP-43 mislocalisation or aggregation (Supplementary Material, Fig. S1), or increase in oxidative stress were observed in the NSC34 (G4C2)102 cells (Supplementary Material, Fig. S2), compared with NSC34 sham cells, at least under basal/unstressed cell culture conditions.

### (G4C2)102 repeat induces cytotoxicity

**Figure 5:**
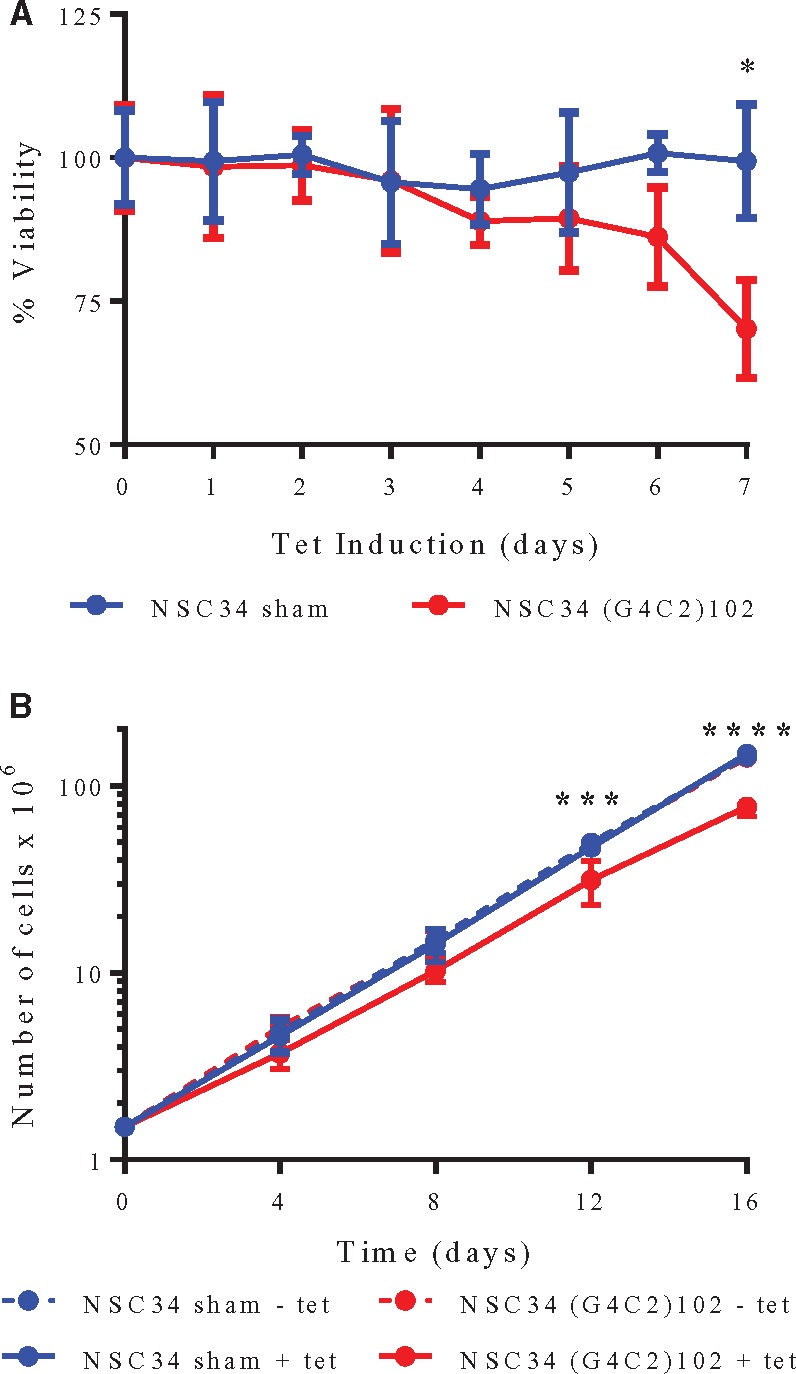
(G4C2)102 repeat is toxic in NSC34 cells. **(A)** NSC34 sham and NSC34 (G4C2)102 cells were cultured for 7 days and were induced for various lengths of time with 0.5 µg/ml tetracycline. Cell viability was measured using an MTT assay (*p < 0.05; two-way ANOVA with Tukey’s multiple comparisons post hoc test; data shown are mean and SD; *n* = 3). **(B)** NSC34 sham and NSC34 (G4C2)102 cells were cultured for 16 days with or without 0.5 µg/ml tetracycline. The cells were counted every 4 days, and then 1.5 × 10^6^ cells were reseeded (***p < 0.001; ****p < 0.0001; two-way ANOVA with Tukey’s multiple comparisons post hoc test; data shown are mean and SD; *n* = 4).

Next, we assessed whether the (G4C2)102 repeat was toxic in the motor neuronal NSC34 cells. The NSC34 cells were grown for 7 days, and induced with tetracycline for increasing lengths of time. The NSC34 (G4C2)102 showed reduced viability of 29.9 ± 8.6% (*p* < 0.05) in an MTT assay after 7 days tetracycline induction compared with NSC34 sham cells. In addition, tetracycline exposure did not reduce viability in the NSC34 sham cells (Fig. 5A). A growth curve was also performed on the NSC34 sham and NSC34 (G4C2)102 cells, with or without 0.5 µg/ml tetracycline (Fig. 5B). Cells were cultured for 16 days in total, viable cells were counted every 4 days and 1.5 × 10^6^ cells were re-seeded and cultured. There were significantly fewer viable NSC34 (G4C2)102 cells when induced with tetracycline after 12 days compared with NSC34 sham cells ± tetracycline and NSC34 (G4C2)102 cells without tetracycline. Specifically, there were only 66.8 ± 26.5% (*p* < 0.001) of NSC34 (G4C2)102 + tetracycline at day 12 and 52.4 ± 11.6% (*p* < 0.0001) at day 16 compared with NSC34 sham + tetracycline (Fig. 5B). In contrast, there were no significant differences in the number of NSC34 sham with or without tetracycline and NSC34 (G4C2)102 cells without tetracycline.

### (G4C2)n affects the PI3K/Akt signalling pathway at transcriptomic level

We had previously undertaken transcriptome analysis on laser captured microdissected (LCM) motor neurons from the spinal cord of C9ORF72-ALS patients (30). Within this dataset, *PTEN* (phosphatase and tensin homolog) had the highest fold change of all differentially expressed genes (fold change = + 11.3, *p* = 0.00001, Supplementary Material, Table 1). Additionally, previous transcriptome analysis on LCM motor neurons from spinal cord motor neurons of SOD1-ALS patients showed that the PI3K/Akt signalling pathway, negatively regulated by PTEN, was increased in the surviving motor neurons, and may provide a therapeutic target (54). Therefore, we investigated the PI3K/Akt signalling pathway in the Kyoto Encyclopaedia of Genes and Genomes (KEGG), in both the human C9ORF72-ALS LCM motor neurons and the murine NSC34 (G4C2)n cells. Careful statistical analysis revealed that this pathway was significantly dysregulated in LCM motor neurons carrying the (G4C2)n C9ORF72 repeat expansion (rank-product, *p* = 0.01), and the NSC34 cells expressing the (G4C2)102 repeat (rank-product, *p*< 0.00001). Transcripts in the PI3K/Akt signalling pathway (KEGG) are listed in Supplementary Tables 1 and 2 for human motor neurons and murine NSC34 cells, respectively.

### PTEN single nucleotide polymorphism (SNP) associated with protection against ALS

We also examined a large genome wide association study (GWAS) using the NeuroX chip (55) to genotype 4890 ALS cases and 5649 normal controls; the NeuroX chip includes genotyping of standard Illumina exome content of approximately 240 000 variants, and additionally, more than 24 000 custom content variants to improve coverage in genes associated with neurological diseases. A list of SNPs found in *PTEN* was identified using BioMart (56). The threshold for significant association with the ALS phenotype was set as a Benjamini–Hochberg-corrected false discovery rate (FDR) < 0.05, which is appropriate for a hypothesis-based rather than a screening-based approach. A single SNP in *PTEN* was associated with risk of ALS (FDR = 0.005). The minor (A) allele of rs202004587 was five times more common in controls than in ALS patients. The minor allele is a missense change which has been associated with hereditary neoplasms by clinical testing (http://www.ncbi.nlm.nih.gov/clinvar/RCV000129085/) and is, therefore, thought to be associated with loss of PTEN function (57). Based on our data, the minor allele should, therefore, be protective against ALS. This further validates the transcriptomic findings from our cell model, and C9ORF72-ALS LCM motor neurons, and suggests that PTEN function could be an important therapeutic target for all ALS. It is possible that the SNP we have identified is in linkage disequilibrium with another more functionally significant variant in the PTEN gene; however, since we are relying on a GWAS dataset with incomplete coverage of the PTEN gene, it is not possible to explore this further with the current data-set.

### Partial Pten knockdown rescues (G4C2)102-induced toxicity

**Figure 6:**
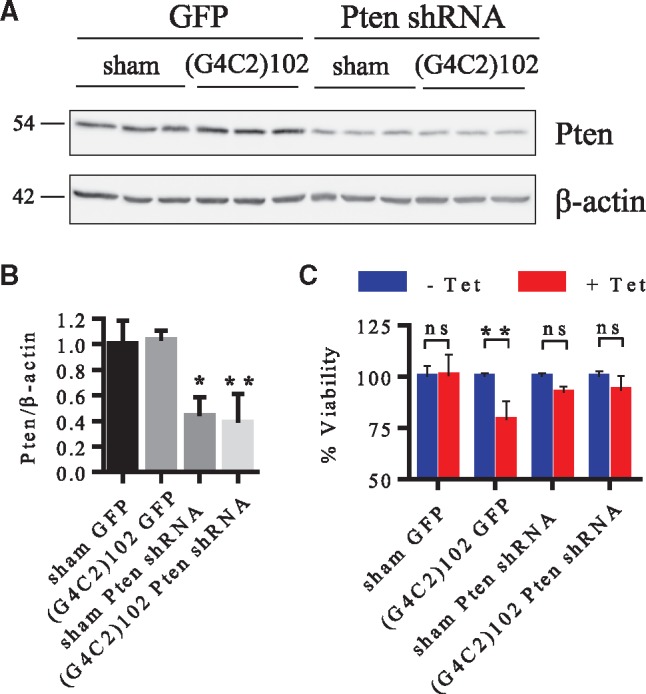
Pten knockdown rescues NSC34 cells from (G4C2)102-induced toxicity. **(A)** Cells were lysed and immunoblotted with anti-Pten. β-Actin was used as a loading control for blots. Molecular weight markers are indicated (kDa). **(B)** Quantification of Pten normalised to β-actin (*p < 0.05; **p < 0.01; one-way ANOVA with Tukey’s multiple comparisons post hoc test; data shown are mean and SD; *n* = 3). **(C)** NSC34 sham GFP, NSC34 (G4C2)102 GFP, NSC34 sham Pten shRNA and NSC34 (G4C2)102 Pten shRNA cells were cultured for 7 days, with (or without) 0.5 µg/ml tetracycline. Cell viability was measured using an MTT assay, and the viability of the tetracycline induced cells was normalised to the non-induced control for each individual cell line (**p < 0.01; two-way ANOVA with Sidak’s multiple comparisons post hoc test; data shown are mean and SD; *n* = 3).

Up-regulation of Pten is consistent with inhibition of the PI3K/Akt signalling pathway. This led us to hypothesise that reducing the expression of Pten in the C9ORF72 *in vitro* model system might rescue the observed (G4C2)n-induced toxicity. NSC34 sham and NSC34 (G4C2)102 were both stably transduced with a lentivirus (LV) expressing either Pten shRNA, or GFP (as a control). Pten was knocked down by 56 ± 7.6% (*p* < 0.05) in the NSC34 sham cells stably transduced with Pten shRNA LV compared with the NSC34 sham cells stably transduced with control GFP LV, and 62.5 ± 22.7% (*p* < 0.01) in the NSC34 (G4C2)102 cells stably transduced with Pten shRNA LV compared with the NSC34 (G4C2)102 cells stably transduced with control GFP LV (Fig. 6A). Additionally, Pten knockdown does not significantly affect the number of RNA foci in the transduced NSC34 (G4C2)102 cells (Supplementary Material, Fig. S4), suggesting that this manipulation does not affect the (G4C2)102 RNA level. The LV transduced NSC34 cells were then grown for 7 days, with or without tetracycline, and viability was assessed. As expected, we detected a significant reduction (21.1 ± 9.1%; *p* < 0.01) in the NSC34 (G4C2)102 GFP LV cell viability when induced with tetracycline (Fig. 6B). However, there was no significant reduction in the NSC34 (G4C2)102 Pten shRNA LV cell viability when induced with tetracycline (Fig. 6B). Therefore, the partial Pten knockdown rescues the NSC34 cells from (G4C2)n-induced toxicity. These results were also replicated using NSC34 sham and (G4C2)102 cells that were transduced with LV expressing scrambled shRNA where the expected cytotoxicity was observed (data not shown). Modulation of PTEN may, therefore, provide a potential therapeutic approach to ameliorate the toxic effects of (G4C2)n-induced toxicity in C9ORF72-ALS.

## Discussion

Recently, various studies have attempted to elucidate the toxic function associated with the (G4C2)n repeat expansion in C9ORF72, which is the most common genetic cause of ALS and FTD (1,2). The repeat expansion is thought to either cause neuronal injury through loss of C9ORF72 function (haploinsufficiency), and/or a gain-of-toxic function from the RNA or DPR proteins. Although there is still much to learn, emerging evidence strongly supports a gain-of-toxic function. First, multiple studies show that the (G4C2)n RNA and/or DPR proteins cause toxicity and/or neurodegeneration in various model systems. Second, while studies interrogating C9ORF72 loss-of-function have reported microglial pathology, they have not reported motor neuron degeneration or TDP-43 pathology associated with C9ORF72 knock down in mice (13,16–21). Third, the targeted knockdown of *C9ORF72* transcripts using ASOs rescues toxicity, and associated transcriptomic changes in cellular models from C9ORF72-ALS/FTD patients (9,20,27).

To investigate the early biochemical changes caused by (G4C2)n repeat expression, we generated stable, isogenic motor neuron-like cell lines with inducible expression of interrupted (G4C2)n repeat constructs. The interrupted (G4C2)102 repeat constructs produced (G4C2)n RNA foci, and underwent RAN translation in the NSC34 (G4C2)n cells, recapitulating key pathological features of C9ORF72-ALS/FTD patient cells. Not surprisingly, there were no antisense-derived RNA foci or RAN translation products in the NSC34 (G4C2)n cells, making this model a sense-only (G4C2)n gain-of-function model. We believe that the new *in vitro* model we have generated will prove useful for screening compounds that modulate the number of RNA foci, the burden of RAN translation products, or the cytotoxicity relating to the presence of the *C9ORF72* G4C2 expansion.

However, while the NSC34 (G4C2)102 cells did recapitulate both the RNA foci and RAN translation of the (G4C2)102 repeat, we did not detect TDP-43 proteinopathy or oxidative stress (two pathological hallmarks of ALS) under basal culture conditions. We did detect colocalisation of the SRSF1, SRSF2 and NCL with the (G4C2)n RNA foci, which is consistent with both C9ORF72-ALS/FTD CNS tissue, and other cellular models expressing the (G4C2)n repeat expansion. Therefore, we reasoned that any biochemical and transcriptomic effects we detected in this cellular model system will represent early changes in response to the presence of the (G4C2)102 repeat. Further, and in agreement with previous studies, the (G4C2)102 repeat reduced NSC34 cell viability and growth rate. However, this could have been caused by either the (G4C2)n RNA foci or the RNA translation proteins produced by the (G4C2)102 transcripts. This is consistent with the literature, showing a gain-of-toxic function associated with the (G4C2)n repeat expansion either through direct RNA toxicity or associated RAN translation protein toxicity (9,20,27,41). Interestingly, however, the proteins generated in this model are hybrids of all sense DPR proteins. There are three possible explanations for the motor neuronal toxicity observed in our model system: (1) the toxicity could be due to the (G4C2)102 RNA alone; (2) the sense DPR motifs may be toxic even when co-occurring in the same polypeptide; (3) the RAN proteins confer toxicity through the poly-GR stretches.

Once we could detect toxicity in the model, the main aim was to use the model for identifying potential therapeutic targets for C9ORF72-ALS/FTD. As a first step towards this goal, we performed transcriptomic analysis on the NSC34 (G4C2)102 cell model. Importantly, using our inducible model system, any changes detected are likely to be the early transcriptomic changes associated with any gain-of-toxic function from the (G4C2)102 repeat. We identified the novel finding that the (G4C2)n repeat dysregulates the PI3K/Akt signalling pathway in a motor neuronal model of C9ORF72-ALS. Further, we validated this finding using transcriptomic analysis of LCM motor neurons from C9ORF72-ALS cases. This is also consistent with previous transcriptome analysis on LCM motor neurons from spinal cord of SOD1-ALS patients (54), implicating the pathway in a broader ALS context. Within the C9ORF72-ALS dataset, *PTEN* had the highest fold change of all DE genes, and previous work has shown PTEN knockdown via siRNA promotes motor neuron survival in ALS and SMA models (54,58,59). Therefore, we partially knocked down Pten in the NSC34 (G4C2)102 cells, which rescued the cells from the (G4C2)102 repeat induced toxicity. Finally, we present evidence from a GWAS study that loss of PTEN function may be protective against ALS more broadly. Taken together, the data we present suggest that PTEN may represent a potential therapeutic target in C9ORF72-ALS, but may also be relevant in other subtypes of ALS.

## Materials and Methods

### Generation of interrupted (G4C2)n repeat constructs

The interrupted (G4C2)102 repeat construct was generated by restriction digest and ligation. Synthesised TCGAC(G4C2)10 sense and ACGT(G2C4)10 antisense ssDNA oligonucleotides (Sigma-Aldrich) were designed so that the dsDNA produced by annealing the oligonucleotides is flanked 5′ by *Sal*I and 3′ by *Xho*I restriction sites. The ssDNA oligos were denatured at 99ºC for 30 min and then annealed by reducing the temperature by 0.5 ºC/min. These (G4C2)10 were ligated into *Sal*I and *Xho*I cut pcDNA6.2-GW/EmGFP-miR (*Invitrogen*), to generate pcDNA6.2-GW/EmGFP-(G4C2)10. Subsequent ligations involved *Xho*I cutting pcDNA6.2-GW/EmGFP-(G4C2)n vectors and inserting further (G4C2)10 repeats. The 5′ *Sal*I site of the inserted (G4C2)n was destroyed whilst the 3′ *Xho*I site was retained – if the insertion orientation was correct. pCMV-EmGFP-(G4C2)102 vectors containing 102 repeats were generated via this method. The exact sequence of the (G4C2)102 construct is described in Figure 1A. EmGFP was subsequently removed from these vectors by *DraI* digestion and religation.

The (G4C2)102 construct was subcloned into pcDNA5/FRT/TO HIS (*Addgene*) using *Dra*I and *Xho*I restriction sites. The FRT/TO/(G4C2)n plasmids were then *Hind*III and *BamH*I cut, Klenow treated and subsequently PNK treated and religated. Transformations of plasmids containing the (G4C2)n repeat constructs were performed using recombination-deficient β-10 *Escherichiacoli* (*NEB*) to minimise (G4C2)n repeat shrinkage. All constructs were checked by sequencing at regular intervals. Plasmids were extracted using a NucleoSpin Plasmid kit (Macherey-Nagel), according to the instructions of the manufacturer.

### Generation of NSC34 (G4C2)102 cell models

Flp-In™ T-REx™ murine motor neuron-like NSC34 cells were generated in house using the Flp-In™ T-REx™ Core Kit (Invitrogen) according to the instructions of the manufacturer. Briefly, an FRT site and tetracycline repressor element were inserted independently into the NSC34 cell genome, and the best clone was selected. Then, pcDNA5/FRT/TO© and pcDNA5/FRT/TO-(G4C2)102 plasmids were stably integrated into the Flp-In™ T-REx™ NSC34 host cell line generating isogenic NSC34 sham and NSC34 (G4C2)102 cell lines, respectively. NSC34 (G4C2)102 cells had tetracycline inducible expression of the inserted (G4C2)102 construct.

### Cell culture, cell line maintenance, plasmid transfection and lentiviral transduction

To maintain the cell lines, the NSC34 sham and NSC34 (G4C2)102 cells were cultured in supplemented DMEM (10% FBS, tetracycline-free (Biosera), 50 U/ml penicillin/streptomycin (Lonza Group Ltd), 100 μg/ml Hygromycin B (*Invitrogen*), 5 μg/ml Blasticidin (Invitrogen) in a 37ºC/5% CO_2_ incubator. Media was replaced every 2–3 days. NSC34 sham and (G4C2)102 cells that were stably transduced with the lentiviral vectors additionally had 1 µg/ml puromycin selection in the supplemented DMEM. HEK293 cells were cultured in supplemented DMEM (10% FBS (*Sigma-Aldrich*), 50 U/ml penicillin/streptomycin) in a 37ºC/5% CO_2_ incubator. NSC34 and HEK293 cells were seeded at an appropriate density, such that the cells were 70-80% confluent at the experimental end point.

For imaging, NSC34 cells were seeded onto gelatin coated coverslips. For each ml of media on the HEK293, 1.4 μg plasmid DNA and 5 μg PEI were diluted in 100 μL OptiMEM (*Life Technologies*), and added dropwise to the media on cells. Cells were incubated for 24 h.

For the lentiviral transduction, NSC34 sham and NSC34 (G4C2)102 cells were transduced with PTEN shRNA (mouse) lentiviral particles (Santa Cruz; sc-36326-V), cop GFP Control lentiviral particles (Santa Cruz; sc-108084), or Control shRNA lentiviral particles (Santa Cruz; sc-108080). Stable transformants were selected for using 1 µg/ml puromycin.

### NSC34 (G4C2)n cell line tetracycline induction

About 0.5 µg/ml tetracycline (*Invitrogen*) was added to NSC34 (G4C2)n cell media, to induce (G4C2)n RNA expression. Tetracycline was added every 3 days (if applicable) to maintain concentration in the media.

### Immunoblotting

NSC34 and HEK293 cells were grown on 6-well plates until 80% confluent. Cells were lysed in lysis buffer (150 mM NaCl, Protease Inhibitor Cocktail EDTA–free (Roche; used to according to the instructions of the manufacturer)) on ice for 20 min. Lysates were centrifuged at 17 000 × *g* at 4 ºC for 5 min to clarify. Supernatant was incubated with Laemmli buffer at 95ºC for 5 min. 25 μg protein from NSC34 lysate and 25 μg protein from HEK293 lysates were separated on 12% or 15% Tris-glycine SDS-polyacrylamide gels and transferred to Amersham™ Protran™ Premium 0.2 nitrocellulose membrane (GE Healthcare). Membranes were blocked in 5% milk (Marvel) in TBS-T at RT for 1 h. Membranes were then probed with mouse anti-GA (1 in 200; kindly provided by Prof Dieter Edbauer), rabbit anti-GP, rabbit anti-GR, rabbit anti-PR, rabbit anti-PA (all 1 in 2000; kindly provided by Prof Stuart Pickering-Brown), rabbit monoclonal anti-PTEN (1 in 1000, Cell Signalling Technology, #9188), mouse monoclonal anti-β actin (1 in 10 000, Abcam, AC-15), or mouse monoclonal anti-α tubulin (1 in 10 000; Sigma-Aldrich; clone DM1A), in 5% Milk/TBS-T at RT for 1 h. Membranes were washed three times in TBS-T, then probed with goat polyclonal anti-rabbit-IgG HRP (1 in 5000; DAKO) or goat polyclonal anti-mouse-IgG HRP (1 in 5000; *DAKO*) in 5% Milk/TBT at RT for 1 h. Membranes were washed three times in TBS-T. Bands were visualised with EZ ECL chemiluminescence detection kit for HRP (Geneflow Ltd) according to the instructions of the manufacturer and imaged using a G-BOX (Syngene).

### RNA fish

A 5’ TYE-563-labelled LNA (16-mer fluorescent)-incorporated DNA probe was used against the sense and antisense RNA hexanucleotide repeat (Exiqon Inc., batch numbers 607323 and 515905, respectively). Slides with tissue were fixed in 4% paraformaldehyde for 10 min. Before use, formalin fixed paraffin-embedded tissue sections were deparaffinized. Slides with NSC34 and HEK293 cells were fixed and permeabilised in 4% PFA/0.2% Triton X-100 for 20 min. For the RNAse-treated control, slides were incubated with 10 μg/ml RNAse A at 37ºC for 30 min. Slides were blocked with hybridization solution [50% formamide, 2 × saline sodium citrate (SSC), 100 mg/ml dextran sulphate, 50 mM sodium phosphate pH 7.0] at 66ºC for 1 h and then incubated with 400 ng/ml of denatured probe in hybridization solution at 66 ºC overnight. After hybridization, slides were washed once in 2 × SSC/0.1% Tween-20 at RT for 5 min and three times in 0.1 × SSC at 65ºC for 10 min. NSC34 cells that were subsequently dual stained by immunocytochemistry (ICC) were first irradiated on ice with 0.3 J/cm^2^ UV, washed three times with PBS, and then ICC staining was performed. Slides were mounted with mounting medium containing DAPI (Vector Labs Inc.). All solutions were made with DEPC-treated water.

### Immunocytochemistry and immunohistochemistry

Slides with NSC34 cells were fixed and permeabilised in 4% PFA/0.2% Triton X-100 at RT for 20 min. Slides were incubated with rabbit polyclonal anti-TDP-43 (1 in 200, Proteintech, 10782-2-AP), rabbit polyclonal anti-SRSF1 (1 in 200, Abcam ab38017), mouse monoclonal anti-SRSF2 (1 in 200, Abcam ab11826), or rabbit polyclonal anti-NCL (1:200; Proteintech; 10556-1-AP) in 2% BSA in PBS at RT for 1 h. Slides were washed three times in PBS, then incubated with goat anti-rabbit IgG H&L (1 in 1000; Alexa Fluor® 488; *Abcam*) or goat anti-mouse IgG H&L (1 in 1000; Alexa Fluor® 488; Abcam) in 2% BSA in PBS at RT for 1 h. Formalin fixed paraffin-embedded tissue sections were deparaffinized and mouse anti-NCL (1 in 150 in 5% BSA; Abcam; ab136649) stained slides were first antigen retrieved by heat in trisodium citrate pH 6.5 for 20 min, then completed as for ICC. Slides of NSC34 and tissue sections were mounted with mounting medium containing DAPI (Vector Labs Inc.).

### Microscopy imaging

*RNA* *foci* *and RBP*: RNA foci were visualised using a Leica SP5 confocal microscope system with a X63/1.4 oil immersion objective lens. The presence of foci was assessed within a high-resolution (1433 mm^2^ per image, 511 × 511 pixels) z-stack made up of images at 0.13 µm intervals through the entire nuclear volume of the cell under consideration. RNA foci were quantified manually. The same imaging was used for RNA foci and RBP (SRSF1, SRSF2 and NCL) co-staining, and for each RBP foci co-stain 50 NSC34 (G4C2)102 cells were analysed for co-localisation.

*NCL* *:* NCL staining in the NSC34 cells and tissue was visualised using a Leica SP5 confocal microscope system with an X63/1.4 oil immersion objective lens. (3775 mm^2^ per image, 511 × 511 pixels) z-stack made up of images at 0.5-mm intervals through the entire nuclear volume of the cell under consideration. To quantify the NCL area relative to the nuclear area, we used the analysis previously described by Haeusler *et al.* 2014 (28). Briefly, a threshold of 50–100 was set in FIJI to measure the nucleolar NCL area, relative to the nuclear area (defined by DAPI staining). Data are means ± SD *n* = 3 for NSC34 cells.

*TDP-43*: TDP-43 staining in NSC34 cells were visualised using a Nikon DS Ri1 Eclipse.

### MTT assay

NSC34 cells were grown for 7 days on a 96-well plate. 0.5 µg/ml tetracycline was added to media, and re-added every 3 days. After 7 days incubation, 0.5 µg/ml MTT reagent (Sigma-Aldrich) was added to media. Plates were incubated in a 37ºC/5% CO_2_ incubator for 90 min. One volume SDS/DMF (20% sodium dodecyl sulphate in 50% dimethyl formamide, pH 4.7) was added to lyse cells. An absorbance of 595 nm of wells was assessed using a PHERAstar FS plate reader (BMG Labtech Ltd).

### DCF assay

NSC34 cells were grown on 96-well plates for 5 days. 0.5 µg/ml tetracycline was added to media and re-added every 3 days. After 5 days incubation, media was replaced with supplemented phenol-red free DMEM (Lonza) + 10% FBS, tetracycline free (*Biosera*). Cytosolic reactive oxygen species levels were measured using 6-carboxy-2′,7′-dichlorodihydrofluorescein diacetate, di(acetoxymethyl ester) (DCF; Molecular Probes™, Life Technologies, C-2938) fluorescence. DCF was added to NSC34 cells at 10 µM, and cells were incubated at 37 °C for 90 min. The fluorescence of oxidised DCF was read at Ex485 nm/Em520 nm using a PHERAstar FS plate reader (BMG labtech Ltd). Cells were then freeze-thawed, and cell number was measured by adding 1.5 µM Ethidium homodimer-1 (EthD1, Molecular Probes™, Life Technologies) to the medium. Fluorescence was measured at Ex570 nm/Em610 nm. Raw DCF data were then normalised to EthD1 reading of cell number.

### RNA preparation for microarray

*NSC34* *:* Three replicate sets of NSC34 cells were cultured for 5 days with or without tetracycline. Media and tetracycline (where necessary) were replenished every 3 days. RNA was extracted using TRI-Reagent® (Zymo Research) and a Direct-zol™ RNA MiniPrep kit (Zymo Research) according to the instructions of the manufacturer. RNA quantity and quality was assessed on the Nanodrop spectrophotometer (Thermofisher) and a Bioanalyser (Agilent), respectively, to ensure all samples were of comparable and sufficient quality to proceed.

*Laser capture microdissected (LCM) Motor Neuron Microarray* *:* Refer to Cooper-Knock *et al.* 2015 (30).

### Microarray hybridisation

*NSC34* *:* Total RNA (500 ng) was linearly amplified using the GeneChip® WT PLUS Reagent kit (Affymetrix) according to the instructions of the manufacturer, to produce ss-cDNA. ss-cDNA (5.5 µg) was fragmented and labelled with biotin, then applied to the GeneChip® Mouse Whole Transcript 1.0 ST Array (Affymetrix), according to the instructions of the manufacturer. Three chips were used for each NSC34 cell line and tetracycline induction condition. Array washing and staining was performed in the Fluidics Station 400 according to the Affymetrix protocol. Arrays were scanned using the Affymetrix GeneChip® 3000 7G scanner. The Affymetrix Gene Chip Command Console (Affymetrix) was used to monitor scanning and to convert the raw image file into a cell intensity file (‘.CEL’).

*LCM Motor Neurons* *:* Refer to Cooper-Knock *et al.* 2015 (30). RNA (20–25 ng) was linearly amplified using the Affymetrix Two Cycle cDNA synthesis protocol to produce biotin-labelled copy RNA. Copy RNA (15 μg) was fragmented for 15 min and hybridized to the Human Genome U133 Plus 2.0 GeneChips, according to Affymetrix protocols. Array washing and staining was performed as above.

### Gene expression data analysis

*NSC34* :For the mouse microarrays, RMA normalisation was performed. 61 transcripts within the murine PI3K/Akt signalling pathway (KEGG) were measured on the Mouse Whole Transcript microarray platform in the NSC34 cells (Supplementary Material, Table S2). PCA analysis without statistical manipulation revealed a distinct separation between NSC34 (G4C2)102 and NSC34 sham cells (Supplementary Material, Fig. S3). In order to interrogate this signal further, expression of all transcripts was ranked according to *t*-statistic in a comparison between NSC34 (G4C2)102 and NSC34 sham cell lines. The combined rank-product (60) of transcripts from the PI3K/Akt signalling pathway was compared to 100 000 random permutations of the same number of transcripts. In short, the product of (rank _transcript X/_total number of transcripts) was calculated where transcript X was each member of the PI3K/Akt pathway in turn; then the probability that this rank-product occurred by ‘chance’ was determined by comparison with random selections of the same number of transcripts where each rank is equally probable. Differential expression of individual transcripts (Supplementary Material, Table S2) was examined by t-test and fold change.

*LCM Motor Neurons* *:* Data from the laser captured microdissection motor neurons were normalised using the *PUMA* package (61,62). Around 283 transcripts within the human PI3K/Akt signalling pathway (KEGG) were measured on the Human U133-Plus 2.0 microarray platform in the laser-captured motor neurons (Supplementary Material, Table S1). Expression of all transcripts was ranked according to *t*-statistic in a comparison between motor neurons with and without expansion of C9ORF72. The combined rank-product (60) of transcripts from the PI3K/Akt signalling pathway was compared with 100 000 random permutations of the same number of transcripts. Differential expression of individual transcripts (Supplementary Material, Table S1) was examined by *t*-test and fold change.

### GWAS analysis

GWAS data were examined using PLINK (http://pngu.mgh.harvard.edu/purcell/plink/ (63)). Genomes were screened from 4890 ALS patients and 5191 controls including 5649 males and 4432 females. All genomes were founders i.e. no two individuals were related to each other. Association analysis was performed and adjusted for multiple testing using the Benjamini and Benjamini & Hochberg (1995) step-up false discovery rate (FDR) control (64). At a significance level of FDR < 0.05, rs202004587 was significantly associated with risk of ALS. Repeating this analysis for a random selection of 500 genes, with approximately the same number of SNPs as PTEN, further suggested that this finding was significant (*p* < 0.05).

## Supplementary Material

Supplementary Material is available at *HMG* online.

## Supplementary Material

Supplementary Data
