# Supplementary material for: C9ORF72 hexanucleotide repeat exerts toxicity in a stable, inducible motor neuronal cell model, which is rescued by partial depletion of Pten
Source: Hum Mol Genet. 2017 Feb 1;26(6):1133–45. doi: 10.1093/hmg/ddx022 (PMC5409131; doi:10.1093/hmg/ddx022)
Supplement: Supplementary Data [file ddx022_Supp.docx]

**Supplementary Materials**

**Members of the NeuroX consortium are:**

Robert Bowser (Division of Neurology, Barrow Neurological Institute), Maura Brunetti (Molecular Genetics Unit, Department of Clinical Pathology, Azienda Sanitaria Ospedaliera Ospedale Infantile Regina Margherita-Santa Anna, Turin), Luigi Ferrucci (Longitudinal Studies Section, Clinical Research Branch, National Institute on Aging, National Institutes of Health, Baltimore) Pietro Fratta (Department of Neurodegenerative Disease, University College London, London), John Hardy (Department of Molecular Neuroscience and Reta Lila Weston Laboratories, Institute of Neurology, University College London, London), Hannu Laaksovirta (Department of Neurology, Helsinki University Central Hospital and Molecular Neurology Programme, Biomedicum, University of Helsinki, Helsinki), Francesco Landi (Department of Gerontology, Geriatrics, and Rehabilitative Medicine, Catholic University of Sacred Heart, Rome), Nicholas Maragakis (Brain Science Institute, Department of Neurology, Johns Hopkins University, Baltimore), Michael Nalls (Molecular Genetics Section, Laboratory of Neurogenetics, National Institute on Aging, National Institutes of Health, Bethesda), Richard Orrell (Department of Clinical Neuroscience, Institute of Neurology, University College London, London), Lyle Ostrow (Brain Science Institute, Department of Neurology, Johns Hopkins University, Baltimore), Gabriella Restagno (Molecular Genetics Unit, Department of Clinical Pathology, Azienda Sanitaria Ospedaliera Ospedale Infantile Regina Margherita-Santa Anna, Turin), Ekaterina Rogaeva (Tanz Centre for Research of Neurodegenerative Diseases, Division of Neurology, Department of Medicine, University of Toronto, Toronto), Jeffrey Rothstein (Brain Science Institute, Department of Neurology, Johns Hopkins University, Baltimore), Andrew Singleton (Molecular Genetics Section, Laboratory of Neurogenetics, National Institute on Aging, National Institutes of Health, Bethesda) Pentti Tienari (Department of Neurology, Helsinki University Central Hospital and Molecular Neurology Programme, Biomedicum, University of Helsinki, Helsinki), Juan Troncoso (Department of Neuropathology, Johns Hopkins University Baltimore), Lorne Zinman (Division of Neurology, Department of Internal Medicine, Sunnybrook Health Sciences Centre, University of Toronto, Toronto), Rita Guerreiro (Department of Molecular Neuroscience, UCL Institute of Neurology, London), Jose Bras (Department of Molecular Neuroscience, UCL Institute of Neurology, London), John Powell (King's College London, Institute of Psychiatry, Department of Neuroscience, London), Michelle K. Lupton (King's College London, Institute of Psychiatry, Department of Neuroscience, London and QIMR Berghofer Medical Research Institute, Genetic Epidemiology, Brisbane), Katie Lunnon (University of Exeter Medical School, RILD, Exeter), Olaf Ansorge (Department of Neuropathology, Nuffield Department of Clinical Neurosciences, University of Oxford, John Radcliffe Hospital), Laura Parkkinen (Oxford Parkinson's Disease Centre, Nuffield Department of Clinical Neurosciences, University of Oxford).

**Members of the ITALSGEN consortium are:**

Francesco O. Logullo (Ancona); Isabella Simone (Bari); Giancarlo Logroscino (Bari and Tricase, LE); Fabrizio Salvi, Ilaria Bartolomei (Bologna); Giuseppe Borghero, Maria Rita Murru, Emanuela Costantino, Carla Pani, Roberta Puddu, Carla Caredda, Valeria Piras, Stefania Tranquilli, Stefania Cuccu, Daniela Corongiu, Maurizio Melis, Antonio Milia, Francesco Marrosu, Maria Giovanna Marrosu, Gianluca Floris, Antonino Cannas, Stefania Cuccu, Stefania Tranquilli (Cagliari); Margherita Capasso (Chieti); Claudia Caponnetto, Gianluigi Mancardi, Paola Origone, Paola Mandich (Genova); Francesca L. Conforti (Mangone, CS); Gabriele Mora, Kalliopi Marinou, Riccardo Sideri (Milan, IRCCS Maugeri Foundation); Silvana Penco, Lorena Mosca (Milan, Niguarda Ca' Granda Hospital); Christian Lunetta (NeuroMuscular OmniCenter, NEMO, Milan); Giuseppe Lauria Pinter (Milan, Besta Neurological Institute), Massimo Corbo (Milan, Casa di Cura del Policlinico); Nilo Riva, Paola Carrera (Milan, IRCCS San Raffaele Scientific Institute); Paolo Volanti (Mistretta, ME); Jessica Mandrioli, Nicola Fini, Antonio Fasano (Modena); Lucio Tremolizzo, Alessandro Arosio, Carlo Ferrarese (Monza); Francesca Trojsi, Gioacchino Tedeschi, Maria Rosaria Monsurrò, Giovanni Piccirillo, Cinzia Femiano (Napoli); Anna Ticca (Nuoro); Enzo Ortu (Ozieri); Vincenzo La Bella, Rossella Spataro, Tiziana Colletti (Palermo); Mario Sabatelli, Marcella Zollino, Amelia Conte, Marco Luigetti, Serena Lattante, Giuseppe Marangi (Rome, Catholic University of Sacred Heart); Marialuisa Santarelli (Rome, San Filippo Neri Hospital); Antonio Petrucci (Rome, San Camillo Forlanini Hospital); Maura Pugliatti, Angelo Pirisi, Leslie D. Parish, Patrizia Occhineri (Sassari), Fabio Giannini, Stefania Battistini, Claudia Ricci, Michele Benigni (Siena); Tea B. Cau, Daniela Loi (Tempio-Olbia); Andrea Calvo, Cristina Moglia, Maura Brunetti, Marco Barberis, Gabriella Restagno, Federico Casale, Giuseppe Marrali, Giuseppe Fuda, Irene Ossola, Stefania Cammarosano, Antonio Canosa, Antonio Ilardi, Umberto Manera, Davide Bertuzzo (Torino), Raffaella Tanel (Trento); Fabrizio Pisano (Veruno, NO).


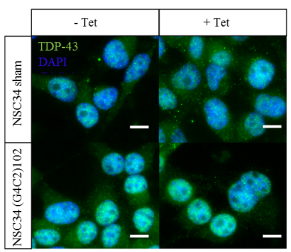


**Fig. S1.** NSC34 (G4C2)102 cells do not show TDP-43 mislocalisation. NSC34 sham and (G4C2)102 cells were cultured with or without 0.5 µg/mL tetracycline for 9 days. Cells were stained for TDP-43 (Green) and Dapi (Blue). Scale bar = 10 µm.


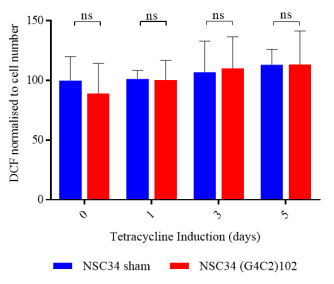


**Fig. S2.** NSC34 (G4C2)102 cells do not show signs of oxidative stress. NSC34 sham and (G4C2)102 cells were cultured for 5 days, and were induced for various lengths of time with 0.5 µg/mL tetracycline. The level of reactive oxygen species (ROS) in the NSC34 cells was measured using the DCF assay, and normalised to cell number (measured after cell lysis using EthD1 fluorescence assay). (Two-way ANOVA with Tukey’s post hoc test; data shown are mean and SD; n = 3).


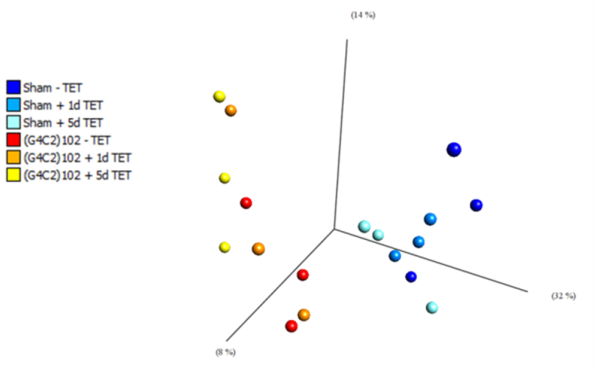


**Fig. S3.** PCA analysis of NSC34 sham and NSC34 (G4C2)102 cells without statistical manipulation.

| Transcript ID | P-value | Fold Change | Log2 Fold Change | Gene Symbol |
| --- | --- | --- | --- | --- |
| 228006_at | 1.35E-05 | 11.25456 | 3.49243775 | PTEN |
| 201125_s_at | 0.001193 | 5.584296 | 2.481375415 | ITGB5 |
| 212777_at | 0.019792 | 3.356961 | 1.747155776 | SOS1 |
| 209341_s_at | 0.116312 | 3.168838 | 1.663953907 | IKBKB |
| 202686_s_at | 0.012691 | 3.109265 | 1.636573582 | AXL |
| 201124_at | 0.023576 | 2.868891 | 1.520493156 | ITGB5 |
| 213093_at | 0.00332 | 2.802556 | 1.486743201 | PRKCA |
| 226731_at | 0.0494 | 2.734129 | 1.451081313 | ITGA1 |
| 203685_at | 0.064852 | 2.704587 | 1.435408306 | BCL2 |
| 202723_s_at | 0.027603 | 2.606658 | 1.38220131 | FOXO1 |
| 203809_s_at | 0.078663 | 2.553882 | 1.352691868 | AKT2 |
| 210482_x_at | 0.143572 | 2.434591 | 1.283679427 | MAP2K5 |
| 1555612_s_at | 0.06008 | 2.428381 | 1.27999479 | G6PC |
| 1552610_a_at | 0.189486 | 2.334724 | 1.223252011 | JAK1 |
| 217399_s_at | 0.164479 | 2.210499 | 1.144372082 | FOXO3 |
| 202887_s_at | 0.000448 | 2.168607 | 1.116768628 | DDIT4 |
| 223196_s_at | 0.090703 | 2.102367 | 1.072014536 | SESN2 |
| 208536_s_at | 0.336397 | 2.082498 | 1.05831511 | BCL2L11 |
| 203379_at | 0.143807 | 2.082037 | 1.057995707 | RPS6KA1 |
| 226068_at | 0.077805 | 2.037049 | 1.026480684 | SYK |
| 202284_s_at | 0.164219 | 2.018647 | 1.013388649 | CDKN1A |
| 225691_at | 0.079002 | 2.010563 | 1.007599543 | CDK12 |
| 236664_at | 0.277998 | 2.005314 | 1.003828157 | AKT2 |
| 201834_at | 0.326416 | 2.005067 | 1.003650446 | PRKAB1 |
| 202830_s_at | 0.148225 | 2.001074 | 1.000774519 | SLC37A4 |
| 212590_at | 0.152239 | 1.874144 | 0.906231807 | RRAS2 |
| 232068_s_at | 0.204519 | 1.867146 | 0.900834742 | TLR4 |
| 222999_s_at | 0.045031 | 1.829493 | 0.871443896 | CCNL2 |
| 201739_at | 0.016888 | 1.828987 | 0.871044821 | SGK1 |
| 202340_x_at | 0.348162 | 1.82347 | 0.866686464 | NR4A1 |
| 202530_at | 0.429409 | 1.769648 | 0.823462423 | MAPK14 |
| 209666_s_at | 0.283035 | 1.739357 | 0.798554074 | CHUK |
| 226441_at | 0.095141 | 1.715889 | 0.778956229 | MAP3K2 |
| 204054_at | 0.392798 | 1.693505 | 0.760012246 | PTEN |
| 202431_s_at | 0.401722 | 1.67664 | 0.745572954 | MYC |
| 202847_at | 0.372926 | 1.671302 | 0.740972448 | PCK2 |
| 221060_s_at | 0.50849 | 1.666396 | 0.736731281 | TLR4 |
| 236459_at | 0.245049 | 1.629361 | 0.704306282 | PRKCE |
| 224889_at | 0.512673 | 1.627228 | 0.702416409 | FOXO3 |
| 209342_s_at | 0.381914 | 1.612545 | 0.689339421 | IKBKB |
| 239201_at | 0.227475 | 1.597199 | 0.675544074 | CDK15 |
| 1558143_a_at | 0.05218 | 1.576673 | 0.656883478 | BCL2L11 |
| 204297_at | 0.245594 | 1.573533 | 0.654007435 | PIK3C3 |
| 202853_s_at | 0.006202 | 1.559587 | 0.641164034 | RYK |
| 210449_x_at | 0.501364 | 1.558745 | 0.640384932 | MAPK14 |
| 212589_at | 0.333773 | 1.554202 | 0.636174023 | RRAS2 |
| 212628_at | 0.240387 | 1.535592 | 0.618794949 | PKN2 |
| 208712_at | 0.329863 | 1.512912 | 0.597328074 | CCND1 |
| 209364_at | 0.437234 | 1.493794 | 0.578981209 | BAD |
| 206398_s_at | 0.490597 | 1.488985 | 0.57432922 | CD19 |
| 202449_s_at | 0.366843 | 1.442837 | 0.528908325 | RXRA |
| 202426_s_at | 0.556319 | 1.440839 | 0.526909137 | RXRA |
| 241453_at | 0.417711 | 1.431025 | 0.517048876 | PTK2 |
| 207540_s_at | 0.466116 | 1.425089 | 0.511052022 | SYK |
| 201234_at | 0.506568 | 1.42441 | 0.510364469 | ILK |
| 241387_at | 0.440439 | 1.417247 | 0.503091215 | PTK2 |
| 225066_at | 0.269049 | 1.401803 | 0.487283617 | PPP2R2D |
| 241722_x_at | 0.557553 | 1.38925 | 0.47430624 | MCL1 |
| 212332_at | 0.25134 | 1.352265 | 0.435377901 | RBL2 |
| 208824_x_at | 0.057664 | 1.346003 | 0.428681625 | CDK16 |
| 208823_s_at | 0.337467 | 1.336739 | 0.418717805 | CDK16 |
| 200797_s_at | 0.166945 | 1.333536 | 0.415256772 | MCL1 |
| 227073_at | 0.507719 | 1.317354 | 0.397643079 | MAP3K2 |
| 225690_at | 0.513019 | 1.306153 | 0.385323901 | CDK12 |
| 206854_s_at | 0.567931 | 1.285591 | 0.362431734 | MAP3K7 |
| 226310_at | 0.425082 | 1.283579 | 0.360172092 | RICTOR |
| 211333_s_at | 0.610405 | 1.280122 | 0.356281311 | FASLG |
| 206952_at | 0.527055 | 1.279746 | 0.355857497 | G6PC |
| 206853_s_at | 0.338928 | 1.268168 | 0.342745879 | MAP3K7 |
| 208820_at | 0.335075 | 1.255543 | 0.328311439 | PTK2 |
| 202161_at | 0.679044 | 1.23813 | 0.308162801 | PKN1 |
| 209184_s_at | 0.246192 | 1.236105 | 0.305801297 | IRS2 |
| 202210_x_at | 0.596252 | 1.235289 | 0.304848605 | GSK3A |
| 219226_at | 0.714163 | 1.230401 | 0.299128581 | CDK12 |
| 211087_x_at | 0.610452 | 1.226702 | 0.294784821 | MAPK14 |
| 211561_x_at | 0.609939 | 1.21891 | 0.285591606 | MAPK14 |
| 202724_s_at | 0.764604 | 1.218777 | 0.28543418 | FOXO1 |
| 212312_at | 0.705952 | 1.208077 | 0.272712412 | BCL2L1 |
| 215195_at | 0.710539 | 1.20301 | 0.266648635 | PRKCA |
| 201984_s_at | 0.428889 | 1.201119 | 0.264379092 | EGFR |
| 225606_at | 0.705461 | 1.199045 | 0.261885804 | BCL2L11 |
| 204906_at | 0.331858 | 1.197615 | 0.260164196 | RPS6KA2 |
| 229705_at | 0.032404 | 1.189092 | 0.249860341 | PIK3C3 |
| 205271_s_at | 0.806096 | 1.187002 | 0.247322366 | CDK20 |
| 207163_s_at | 0.426152 | 1.186063 | 0.246180643 | AKT1 |
| 1569272_at | 0.674759 | 1.179942 | 0.238715946 | PIK3C3 |
| 235254_at | 0.753921 | 1.169931 | 0.226423445 | MAP3K2 |
| 223195_s_at | 0.710065 | 1.155934 | 0.209059027 | SESN2 |
| 214265_at | 0.557851 | 1.13662 | 0.184750006 | ITGA8 |
| 204924_at | 0.772744 | 1.132702 | 0.179768356 | TLR2 |
| 228177_at | 0.828615 | 1.12985 | 0.176131252 | CREBBP |
| 204131_s_at | 0.513469 | 1.12735 | 0.172935488 | FOXO3 |
| 1556655_s_at | 0.868312 | 1.124945 | 0.169854468 | CDK12 |
| 231017_at | 0.600747 | 1.122861 | 0.167179346 | STK11 |
| 228248_at | 0.756222 | 1.117075 | 0.159726051 | RICTOR |
| 202160_at | 0.393015 | 1.115457 | 0.1576349 | CREBBP |
| 238733_at | 0.789257 | 1.10799 | 0.147944861 | MDM2 |
| 226979_at | 0.575744 | 1.102505 | 0.140785199 | MAP3K2 |
| 203984_s_at | 0.783146 | 1.094592 | 0.130393218 | CASP9 |
| 205386_s_at | 0.873715 | 1.092642 | 0.127820785 | MDM2 |
| 208641_s_at | 0.758982 | 1.087062 | 0.120434226 | RAC1 |
| 240964_at | 0.878754 | 1.086979 | 0.120324068 | PTEN |
| 226156_at | 0.867426 | 1.085308 | 0.118104524 | AKT2 |
| 225471_s_at | 0.86043 | 1.083219 | 0.11532495 | AKT2 |
| 203836_s_at | 0.849016 | 1.078407 | 0.108901766 | MAP3K5 |
| 237451_x_at | 0.718962 | 1.076788 | 0.106734237 | CASP9 |
| 220587_s_at | 0.822453 | 1.07642 | 0.106241102 | MLST8 |
| 202685_s_at | 0.908978 | 1.066797 | 0.093285673 | AXL |
| 211372_s_at | 0.883678 | 1.062037 | 0.086834029 | IL1R2 |
| 227627_at | 0.876863 | 1.04972 | 0.070004558 | SGK3 |
| 225160_x_at | 0.802144 | 1.048663 | 0.068551126 | MDM2 |
| 244616_x_at | 0.909744 | 1.04365 | 0.061637969 | MDM2 |
| 227426_at | 0.870694 | 1.040067 | 0.056676468 | SOS1 |
| 211832_s_at | 0.912271 | 1.039894 | 0.056436477 | MDM2 |
| 203837_at | 0.792837 | 1.036344 | 0.051502965 | MAP3K5 |
| 217492_s_at | 0.924599 | 1.035607 | 0.050476622 | PTEN |
| 1557970_s_at | 0.9432 | 1.033772 | 0.047918032 | RPS6KA2 |
| 212629_s_at | 0.935661 | 1.032482 | 0.04611663 | PKN2 |
| 212719_at | 0.886709 | 1.020152 | 0.028784126 | PHLPP1 |
| 209185_s_at | 0.973334 | 1.00973 | 0.01396957 | IRS2 |
| 226299_at | 0.985456 | 1.008872 | 0.012743145 | PKN3 |
| 202670_at | 0.988163 | 1.007332 | 0.01053925 | MAP2K1 |
| 229711_s_at | 0.968365 | 1.006463 | 0.009294136 | MDM2 |
| 225694_at | 0.994284 | 1.004493 | 0.00646751 | CDK12 |
| 632_at | 0.9922 | 1.003997 | 0.005754958 | GSK3A |
| 224891_at | 0.994465 | 1.001816 | 0.002617558 | FOXO3 |
| 208640_at | 0.996 | 1.001424 | 0.002052936 | RAC1 |
| 207239_s_at | 0.999889 | 1.000062 | 8.94443E-05 | CDK16 |
| 213012_at | 0.959725 | 0.981467 | -0.026988334 | NEDD4 |
| 223049_at | 0.907224 | 0.980561 | -0.028320713 | GRB2 |
| 204756_at | 0.936182 | 0.974529 | -0.037222977 | MAP2K5 |
| 215394_at | 0.955286 | 0.970561 | -0.043109205 | PIK3C3 |
| 224999_at | 0.937243 | 0.968813 | -0.045709871 | EGFR |
| 205798_at | 0.938054 | 0.964411 | -0.052279989 | IL7R |
| 212331_at | 0.890071 | 0.96321 | -0.054077725 | RBL2 |
| 232876_at | 0.931585 | 0.959169 | -0.060143063 | MAPK14 |
| 201983_s_at | 0.924495 | 0.95877 | -0.060743327 | EGFR |
| 1555804_a_at | 0.894588 | 0.950584 | -0.073113976 | MAP3K19 |
| 205498_at | 0.898394 | 0.949704 | -0.074450165 | GHR |
| 200980_s_at | 0.803407 | 0.946482 | -0.079353026 | PDHA1 |
| 226218_at | 0.908871 | 0.945496 | -0.08085674 | IL7R |
| 211808_s_at | 0.891156 | 0.940032 | -0.089218226 | CREBBP |
| 202288_at | 0.894346 | 0.931247 | -0.102764222 | MTOR |
| 200798_x_at | 0.772837 | 0.928812 | -0.106541483 | MCL1 |
| 209112_at | 0.594059 | 0.921765 | -0.117529106 | CDKN1B |
| 214328_s_at | 0.75804 | 0.915552 | -0.127286267 | HSP90AA1 |
| 208743_s_at | 0.855563 | 0.911983 | -0.132921163 | YWHAB |
| 201389_at | 0.856486 | 0.910729 | -0.134906271 | ITGA5 |
| 208456_s_at | 0.918453 | 0.908647 | -0.138208164 | RRAS2 |
| 212912_at | 0.705757 | 0.906253 | -0.142014229 | RPS6KA2 |
| 204247_s_at | 0.783234 | 0.902163 | -0.148539976 | CDK5 |
| 217718_s_at | 0.693721 | 0.89881 | -0.153911919 | YWHAB |
| 210655_s_at | 0.789241 | 0.897205 | -0.156490434 | FOXO3 |
| 204369_at | 0.845732 | 0.895233 | -0.159664877 | PIK3CA |
| 201452_at | 0.760296 | 0.89119 | -0.166195051 | RHEB |
| 226046_at | 0.835819 | 0.883273 | -0.179068683 | MAPK8 |
| 239300_at | 0.363481 | 0.883176 | -0.179227127 | PIK3C3 |
| 210775_x_at | 0.847061 | 0.881818 | -0.181447169 | CASP9 |
| 225363_at | 0.559081 | 0.877415 | -0.188668724 | PTEN |
| 218852_at | 0.815605 | 0.876718 | -0.189815226 | PPP2R3C |
| 44654_at | 0.501269 | 0.876607 | -0.189997896 | G6PC3 |
| 210969_at | 0.692941 | 0.87551 | -0.191804438 | PKN2 |
| 209390_at | 0.55561 | 0.873355 | -0.195359897 | TSC1 |
| 210211_s_at | 0.74048 | 0.869027 | -0.202527094 | HSP90AA1 |
| 1567458_s_at | 0.433362 | 0.866275 | -0.207103012 | RAC1 |
| 202647_s_at | 0.659401 | 0.863871 | -0.211112201 | NRAS |
| 1560074_at | 0.59213 | 0.859693 | -0.218106536 | PRKCA |
| 243492_at | 0.763459 | 0.852679 | -0.229925369 | THEM4 |
| 214172_x_at | 0.313143 | 0.850473 | -0.23366266 | RYK |
| 201437_s_at | 0.43703 | 0.846859 | -0.239806311 | EIF4E |
| 1552611_a_at | 0.443595 | 0.846177 | -0.240968623 | JAK1 |
| 224985_at | 0.509339 | 0.846049 | -0.241186874 | NRAS |
| 229664_at | 0.386319 | 0.839849 | -0.251798132 | MAPK8 |
| 215037_s_at | 0.609737 | 0.836868 | -0.256928012 | BCL2L1 |
| 217542_at | 0.617322 | 0.831476 | -0.266253473 | MDM2 |
| 204798_at | 0.727843 | 0.826783 | -0.27441937 | MYB |
| 211814_s_at | 0.774846 | 0.826533 | -0.274855673 | CCNE2 |
| 211550_at | 0.625894 | 0.821579 | -0.283528789 | EGFR |
| 1555864_s_at | 0.597819 | 0.820961 | -0.284614407 | PDHA1 |
| 1552559_a_at | 0.646832 | 0.81826 | -0.289368766 | CDK15 |
| 1553118_at | 0.413062 | 0.816151 | -0.293091998 | THEM4 |
| 226312_at | 0.452194 | 0.813619 | -0.297574725 | RICTOR |
| 211938_at | 0.290751 | 0.803602 | -0.31544694 | EIF4B |
| 211937_at | 0.356438 | 0.80297 | -0.316582007 | EIF4B |
| 204053_x_at | 0.560409 | 0.80144 | -0.319333578 | PTEN |
| 201835_s_at | 0.355711 | 0.796135 | -0.328915007 | PRKAB1 |
| 217557_s_at | 0.631736 | 0.794321 | -0.33220595 | MDM2 |
| 200979_at | 0.285155 | 0.792685 | -0.335180418 | PDHA1 |
| 235980_at | 0.266705 | 0.792475 | -0.335562671 | PIK3CA |
| 221759_at | 0.518122 | 0.791827 | -0.336742833 | G6PC3 |
| 214660_at | 0.677173 | 0.775384 | -0.367017129 | ITGA1 |
| 211536_x_at | 0.63566 | 0.768882 | -0.37916589 | MAP3K7 |
| 212688_at | 0.216451 | 0.764708 | -0.387019128 | PIK3CB |
| 209269_s_at | 0.673538 | 0.762087 | -0.391972389 | SYK |
| 215152_at | 0.580627 | 0.755046 | -0.405363554 | MYB |
| 217717_s_at | 0.373991 | 0.753917 | -0.407522391 | YWHAB |
| 217373_x_at | 0.477403 | 0.752907 | -0.409456422 | MDM2 |
| 210865_at | 0.377927 | 0.74328 | -0.428022306 | FASLG |
| 1861_at | 0.551345 | 0.73509 | -0.444007199 | BAD |
| 201453_x_at | 0.337992 | 0.734954 | -0.444274139 | RHEB |
| 232274_at | 0.329023 | 0.73347 | -0.447190136 | CCNL2 |
| 201648_at | 0.248252 | 0.728557 | -0.456886246 | JAK1 |
| 211027_s_at | 0.518768 | 0.726536 | -0.460893809 | IKBKB |
| 217289_s_at | 0.484965 | 0.726016 | -0.461926752 | SLC37A4 |
| 200796_s_at | 0.597017 | 0.725817 | -0.462322247 | MCL1 |
| 211711_s_at | 0.077039 | 0.721442 | -0.47104468 | PTEN |
| 207005_s_at | 0.486609 | 0.720751 | -0.472427162 | BCL2 |
| 221772_s_at | 0.375017 | 0.716974 | -0.480007292 | PPP2R2D |
| 205403_at | 0.491429 | 0.716384 | -0.481194979 | IL1R2 |
| 220357_s_at | 0.604314 | 0.708024 | -0.49812983 | SGK2 |
| 204531_s_at | 0.434037 | 0.702258 | -0.509926941 | BRCA1 |
| 1552734_at | 0.421191 | 0.702117 | -0.510216635 | RICTOR |
| 216976_s_at | 0.534127 | 0.701251 | -0.511997172 | RYK |
| 230573_at | 0.346966 | 0.696043 | -0.52275166 | SGK2 |
| 209953_s_at | 0.289378 | 0.691039 | -0.533160961 | CDC37 |
| 206248_at | 0.262083 | 0.68902 | -0.537382235 | PRKCE |
| 237891_at | 0.466151 | 0.677031 | -0.562706201 | MDM2 |
| 221695_s_at | 0.600041 | 0.673253 | -0.570779342 | MAP3K2 |
| 212780_at | 0.025322 | 0.669516 | -0.578809562 | SOS1 |
| 1565483_at | 0.422473 | 0.662606 | -0.593776828 | EGFR |
| 227633_at | 0.40547 | 0.662048 | -0.594992275 | RHEB |
| 242674_at | 0.173354 | 0.656777 | -0.606524489 | EIF4E |
| 231228_at | 0.339066 | 0.649862 | -0.621794705 | BCL2L1 |
| 206665_s_at | 0.435532 | 0.648406 | -0.625030654 | BCL2L1 |
| 213404_s_at | 0.144982 | 0.646752 | -0.628715485 | RHEB |
| 217620_s_at | 0.336637 | 0.646357 | -0.629596871 | PIK3CB |
| 231854_at | 0.461979 | 0.64623 | -0.629880368 | PIK3CA |
| 226101_at | 0.163161 | 0.646057 | -0.630266639 | PRKCE |
| 214621_at | 0.372588 | 0.641953 | -0.639460419 | GYS2 |
| 215075_s_at | 0.141904 | 0.634678 | -0.65590326 | GRB2 |
| 201436_at | 0.135331 | 0.634276 | -0.656817341 | EIF4E |
| 208711_s_at | 0.455611 | 0.633879 | -0.657720622 | CCND1 |
| 211370_s_at | 0.484713 | 0.632944 | -0.659850233 | MAP2K5 |
| 41657_at | 0.343057 | 0.630361 | -0.665749816 | STK11 |
| 225715_at | 0.252504 | 0.628748 | -0.669446189 | RPTOR |
| 222343_at | 0.362422 | 0.628394 | -0.670258689 | BCL2L11 |
| 232086_at | 0.386168 | 0.626462 | -0.674701094 | PIK3C3 |
| 221427_s_at | 0.04162 | 0.617323 | -0.695902551 | CCNL2 |
| 224341_x_at | 0.361249 | 0.616781 | -0.697169771 | TLR4 |
| 220038_at | 0.391962 | 0.613958 | -0.703788129 | SGK3 |
| 211851_x_at | 0.461197 | 0.611764 | -0.708952883 | BRCA1 |
| 1560359_at | 0.491519 | 0.607575 | -0.718865587 | ITGA1 |
| 1553096_s_at | 0.054393 | 0.605402 | -0.724034654 | BCL2L11 |
| 221180_at | 0.288631 | 0.59205 | -0.756209075 | MAP3K19 |
| 1553088_a_at | 0.108437 | 0.591752 | -0.756935418 | BCL2L11 |
| 211968_s_at | 0.252139 | 0.59163 | -0.757232885 | HSP90AA1 |
| 239188_at | 0.346612 | 0.590814 | -0.759224082 | PPP2R3C |
| 229253_at | 0.073477 | 0.587559 | -0.767194367 | THEM4 |
| 203684_s_at | 0.405007 | 0.584138 | -0.775618855 | BCL2 |
| 242071_x_at | 0.254398 | 0.578616 | -0.789321877 | ITGA8 |
| 206341_at | 0.334766 | 0.578428 | -0.789790704 | IL2RA |
| 211537_x_at | 0.392671 | 0.567562 | -0.817150095 | MAP3K7 |
| 215735_s_at | 0.118292 | 0.564778 | -0.824244203 | TSC2 |
| 211969_at | 0.289239 | 0.556204 | -0.846313975 | HSP90AA1 |
| 226048_at | 0.094545 | 0.544801 | -0.876198743 | MAPK8 |
| 225697_at | 0.061816 | 0.53981 | -0.889476392 | CDK12 |
| 1565484_x_at | 0.360314 | 0.53701 | -0.896979141 | EGFR |
| 1556654_at | 0.163266 | 0.534599 | -0.903470956 | CDK12 |
| 207821_s_at | 0.339514 | 0.532389 | -0.909447332 | PTK2 |
| 210226_at | 0.258313 | 0.531457 | -0.911975126 | NR4A1 |
| 201020_at | 0.081573 | 0.525793 | -0.92743316 | YWHAH |
| 209239_at | 0.119177 | 0.500991 | -0.997143408 | NFKB1 |
| 235011_at | 0.263437 | 0.491702 | -1.024143872 | MAP3K2 |
| 211607_x_at | 0.118234 | 0.48165 | -1.053942929 | EGFR |
| 237718_at | 0.166519 | 0.480893 | -1.056212169 | EIF4E |
| 206923_at | 0.080503 | 0.480587 | -1.057130471 | PRKCA |
| 1552798_a_at | 0.177667 | 0.473998 | -1.077047123 | TLR4 |
| 239092_at | 0.159601 | 0.470743 | -1.086988453 | ITGA8 |
| 240437_at | 0.10184 | 0.457427 | -1.12838657 | CASP9 |
| 1560689_s_at | 0.162751 | 0.451964 | -1.145720232 | AKT2 |
| 210984_x_at | 0.196761 | 0.439456 | -1.18620937 | EGFR |
| 205034_at | 0.015597 | 0.422482 | -1.243038219 | CCNE2 |
| 1554826_at | 0.232229 | 0.416916 | -1.262171355 | CDK15 |
| 211453_s_at | 0.032224 | 0.414763 | -1.269640894 | AKT2 |
| 201435_s_at | 0.089872 | 0.410499 | -1.284549387 | EIF4E |
| 211269_s_at | 0.173879 | 0.403133 | -1.31067221 | IL2RA |
| 1555780_a_at | 0.236837 | 0.39096 | -1.354907085 | RHEB |
| 211297_s_at | 0.031508 | 0.383244 | -1.383664889 | CDK7 |
| 204132_s_at | 0.02473 | 0.360665 | -1.471268668 | FOXO3 |
| 210671_x_at | 0.043001 | 0.357881 | -1.482448142 | MAPK8 |
| 235666_at | 0.063203 | 0.308998 | -1.694330595 | ITGA8 |
| 210477_x_at | 0.029171 | 0.288981 | -1.790953454 | MAPK8 |
| 204292_x_at | 0.007038 | 0.222178 | -2.170212126 | STK11 |

**Table S1.** Differentially expressed transcripts within the human PI3K/Akt signalling pathway (KEGG) measured on the Human Genome microarray platform in the Laser Captured Microdissected motor neurons. Transcript ID, Fold change and p-value are included for a comparison between C9ORF72-ALS patients and control LCM MNs.

| Transcript ID | P-value | Fold Change | Log2 Fold Change | Gene Symbol |
| --- | --- | --- | --- | --- |
| ENSMUST00000061673 | 2.18E-08 | 1.41441 | 0.50020038 | ITGA1 |
| ENSMUST00000146023 | 5.21E-08 | 1.33624 | 0.418179151 | BCL2L11 |
| ENSMUST00000013807 | 4.82E-03 | 1.19164 | 0.252948457 | PTEN |
| ENSMUST00000135810 | 9.37E-05 | 1.18084 | 0.239813497 | PHLPP1 |
| ENSMUST00000061512 | 2.32E-03 | 1.13641 | 0.184483432 | MAP3K19 |
| ENSMUST00000017290 | 3.04E-02 | 1.13287 | 0.179982317 | BRCA1 |
| ENSMUST00000160662 | 4.83E-04 | 1.13155 | 0.178300335 | CDK15 |
| ENSMUST00000027243 | 2.31E-03 | 1.12736 | 0.172948285 | IL1R2 |
| ENSMUST00000033500 | 1.25E-02 | 1.12504 | 0.169976296 | ERAS |
| ENSMUST00000019469 | 1.37E-02 | 1.11416 | 0.155956427 | G6PC |
| ENSMUST00000112751 | 2.62E-04 | 1.10624 | 0.145664414 | BCL2 |
| ENSMUST00000018012 | 1.10E-03 | 1.1019 | 0.139993302 | SGK2 |
| ENSMUST00000019615 | 4.00E-02 | 1.09648 | 0.132879497 | CDC37 |
| ENSMUST00000120135 | 5.31E-03 | 1.08072 | 0.111992789 | SYK |
| ENSMUST00000048096 | 9.17E-02 | 1.07976 | 0.110710678 | TLR4 |
| ENSMUST00000125346 | 1.38E-02 | 1.0793 | 0.110095929 | PKN3 |
| ENSMUST00000020308 | 4.67E-01 | 1.06268 | 0.08770723 | DDIT4 |
| ENSMUST00000129514 | 1.13E-01 | 1.04996 | 0.070334367 | RXRA |
| ENSMUST00000144883 | 3.93E-01 | 1.02521 | 0.035919456 | STK11 |
| ENSMUST00000053764 | 3.96E-01 | 1.02437 | 0.034736907 | FOXO1 |
| ENSMUST00000166384 | 4.79E-01 | 1.01442 | 0.020655095 | SGK3 |
| ENSMUST00000106499 | 9.58E-01 | 1.00188 | 0.00270972 | GRB2 |
| ENSMUST00000094361 | 9.16E-01 | 0.99356 | -0.009321002 | HSP90AA1 |
| ENSMUST00000023779 | 4.55E-01 | 0.98085 | -0.027895571 | NR4A1 |
| ENSMUST00000156857 | 3.03E-01 | 0.97905 | -0.030545555 | TSC1 |
| ENSMUST00000049822 | 9.89E-02 | 0.93663 | -0.094448847 | THEM4 |
| ENSMUST00000023165 | 2.95E-01 | 0.93459 | -0.097594494 | CREBBP |
| ENSMUST00000001780 | 3.63E-02 | 0.91375 | -0.130128594 | AKT1 |
| ENSMUST00000125583 | 5.31E-04 | 0.88349 | -0.17871429 | RPTOR |
| ENSMUST00000107539 | 2.63E-02 | 0.88127 | -0.182344001 | CDK12 |
| ENSMUST00000108344 | 3.26E-05 | 0.87959 | -0.185096893 | AKT2 |
| ENSMUST00000128660 | 3.52E-06 | 0.85127 | -0.232311306 | CASP9 |
| ENSMUST00000115028 | 4.62E-06 | 0.82873 | -0.271025946 | ITGB5 |
| ENSMUST00000114758 | 1.80E-04 | 0.81702 | -0.2915567 | MAPK14 |
| ENSMUST00000031486 | 2.93E-09 | 0.80861 | -0.30648405 | PRKAB1 |
| ENSMUST00000128157 | 9.07E-06 | 0.80835 | -0.306948007 | PPP2R2D |
| ENSMUST00000043812 | 1.66E-04 | 0.80476 | -0.313369496 | PKN2 |
| ENSMUST00000115812 | 4.61E-07 | 0.80411 | -0.314535223 | PIK3C3 |
| ENSMUST00000107739 | 6.30E-08 | 0.79307 | -0.334479884 | ITGA3 |
| ENSMUST00000019109 | 3.23E-06 | 0.78673 | -0.346059497 | YWHAH |
| ENSMUST00000029803 | 6.06E-04 | 0.78327 | -0.352418392 | EIF4E |
| ENSMUST00000110036 | 2.59E-04 | 0.78045 | -0.357621887 | PTK2 |
| ENSMUST00000030724 | 7.98E-07 | 0.78036 | -0.357788265 | SESN2 |
| ENSMUST00000095806 | 1.54E-09 | 0.77172 | -0.3738506 | MAP3K5 |
| ENSMUST00000005066 | 2.95E-09 | 0.75827 | -0.399216449 | MAP2K1 |
| ENSMUST00000093962 | 1.15E-07 | 0.74789 | -0.419102001 | CCND1 |
| ENSMUST00000108243 | 1.70E-05 | 0.74781 | -0.419256331 | PIK3CA |
| ENSMUST00000070334 | 8.98E-07 | 0.74624 | -0.422288401 | G6PC3 |
| ENSMUST00000097275 | 2.89E-10 | 0.74093 | -0.432590846 | PRKCE |
| ENSMUST00000037607 | 7.29E-06 | 0.73346 | -0.447209806 | MAP3K7 |
| ENSMUST00000018470 | 3.42E-05 | 0.73286 | -0.448390472 | YWHAB |
| ENSMUST00000071739 | 3.92E-06 | 0.72087 | -0.472188984 | GSK3A |
| ENSMUST00000037947 | 1.34E-06 | 0.71222 | -0.489605146 | MCL1 |
| ENSMUST00000175883 | 9.14E-08 | 0.70524 | -0.503813791 | RYK |
| ENSMUST00000115364 | 1.06E-09 | 0.67775 | -0.561174887 | CDK16 |
| ENSMUST00000111945 | 1.34E-08 | 0.66852 | -0.580957373 | MAPK8 |
| ENSMUST00000034740 | 7.86E-06 | 0.64571 | -0.631041725 | NEDD4 |
| ENSMUST00000145430 | 9.93E-07 | 0.63284 | -0.660087304 | CCNE2 |
| ENSMUST00000028106 | 8.25E-07 | 0.61691 | -0.696868063 | ITGA8 |
| ENSMUST00000119901 | 1.53E-07 | 0.54694 | -0.870545519 | CDKN1A |
| ENSMUST00000033662 | 6.12E-10 | 0.50029 | -0.999163479 | PDHA1 |

**Table S2.** Differentially expressed transcripts within the murine PI3K/Akt signalling pathway (KEGG) measured on the Mouse Transcriptome microarray platform in the NSC34 cells. Probe ID, Fold change and p-value are included for a comparison between NSC34 (G4C2)102 and NSC34 sham cells.


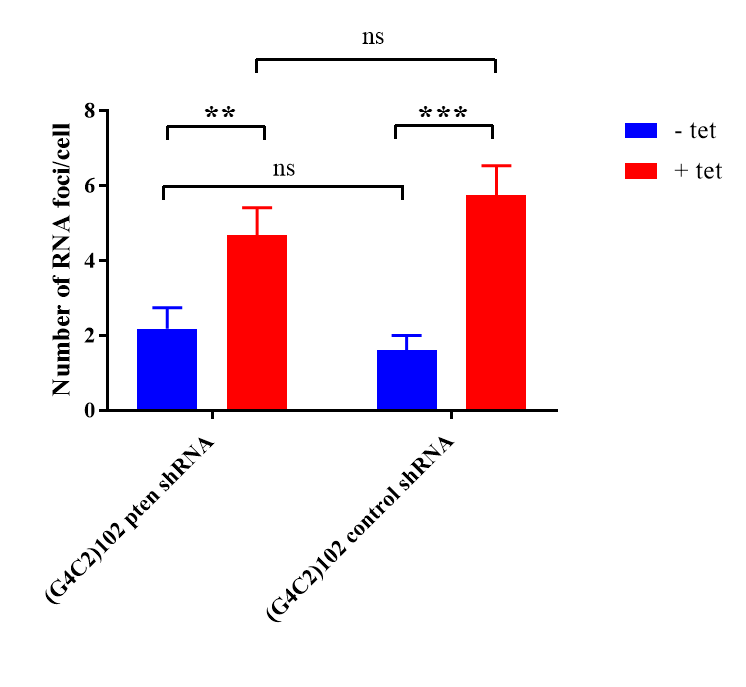


**Fig. S4.** Pten knockdown does not affect the number of (G4C2)n RNA foci. NSC34 (G4C2)102 control shRNA and NSC34 (G4C2)102 Pten shRNA cells were cultured for 3 days, with (or without) 0.5 µg/mL tetracycline. Cells were stained with a Locked Nucleic Acid (C4G2)3 sense probe and Dapi, and the average number of RNA foci per cell was counted (**P<0.01; ***P < 0.001; Two-way ANOVA with Tukey’s multiple comparisons post hoc test; data shown are mean and SD; n = 3).
